# In Situ Fixation of Symptomatic Fibrous Non-union Hoffa Fracture: A Case Report

**DOI:** 10.5704/MOJ.1903.014

**Published:** 2019-03

**Authors:** A Soni, R Kansay, S Gupta, A Malhotra

**Affiliations:** Department of Orthopaedics, Government Medical College and Hospital, Chandigarh, India

**Keywords:** hoffa, non-union, femoral condyle, coronal fracture

## Abstract

Femoral condyle fracture in coronal plane, also known as Hoffa fracture, is a rare fracture. Non-union of Hoffa fracture is even rarer. We present a case of fibrous nonunion of a Hoffa fracture in which the fractured fragment, though not freely movable, led to painful walking. Since the fragment was un-displaced and non-movable we fixed the fractured fragment *in situ*. Patient regained full range of motion of the knee and was asymptomatic on follow-up.

## Introduction

Coronal plane fracture of the femoral condyle, known as Hoffa fracture, is a rare injury. Being an intra-articular fracture, open reduction and rigid fixation is the treatment of choice as non-operative treatment leads to poor outcome. Non-union of Hoffa fracture is even rarer and only a few cases have been reported in the literature^1,2^. We present here an unusual case of un-displaced fibrous non-union of Hoffa fracture. The patient was symptomatic and we fixed the fracture *in situ* with screws.

## Case Report

A 31-year old male patient presented to us with pain right knee while walking and running. He had history of a road traffic accident one-year back following which he had pain and swelling of the right knee. He had been taken to a local hospital. Radiographs were found to be normal and patient was given splintage for three weeks. After three weeks the splint was removed and knee bending started. The pain was found to have decreased but still present while walking. The patient had consulted traditional medicine practitioners and physiotherapists but the problem did not resolve with time. Finally, patient came to our hospital.

**Fig. 1:**
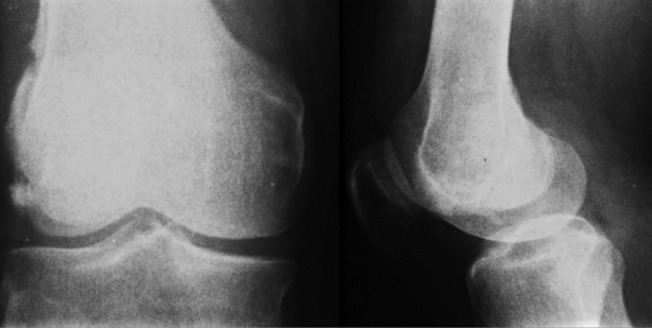
Pre-operative radiograph showing Hoffa fracture.

**Fig. 2:**
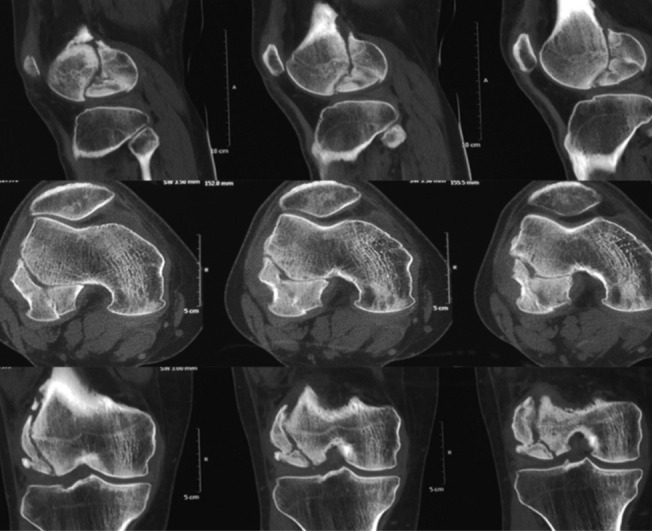
Pre-operative CT scan showing Hoffa fracture of lateral condyle.

On examination in our clinic there was tenderness over the lateral femoral condyle. Range of motion at knee was full and pain free. There was no instability or clunking. Radiographs and computed tomography (CT) scan revealed undisplaced non-union of a Hoffa fracture of the lateral femoral condyle (Fig. 1, Fig. 2). We decided to fix the fracture along with bone grafting at fracture site.

**Fig. 3:**
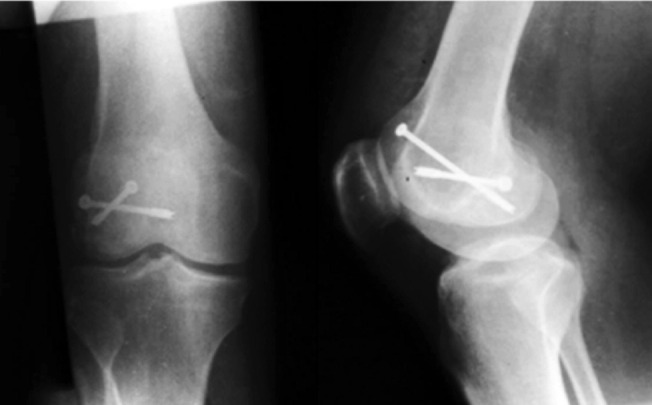
Radiographs at 12 months follow-up showing fixation of fracture with two screws.

We accessed the lateral condyle femur through a lateral approach but intra-operatively we were not able to appreciate the fracture site. There was no abnormal mobility at the site where fracture was seen on the CT scan. We tried manipulating the fractured fragment but there was no movement of the fragment, instead cancellous bone started breaking. We decided that it was fibrous non-union and fixed the fracture *in situ* with two partially threaded screws which were countersunk (Fig. 3). Knee bending and full weight bearing walking was started on post-operative day one. There was no pain on knee bending or walking. At the last follow-up at two years the patient had full range of motion of the knee joint (00-1400) without any pain while walking or running.

## Discussion

The Hoffa fracture is an intra-articular fracture of femoral condyle in the coronal plane, first described by Hoffa in 19043. Though the exact mechanism is not known, it is widely believed that the fracture results from a shear force on the posterior femoral condyle when the knee flexes more than 90 degree^4^. The lateral condyle is more prone because of the natural valgus of the knee joint. In our case the lateral femoral condyle was involved. Undisplaced Hoffa fracture can often be missed on standard anteroposteror and lateral radiographs and that could be a reason for initial missing of the fracture at the first hospital. CT scan can be helpful in such suspicious cases and is recommended^5^.

There are a few cases reported in the literature of non-union of Hoffa fracture, however, no fibrous non-union has been described. McDonough and Bernstein described non-union of Hoffa fracture in an 8-year old child following a road traffic accident^1^. The freely mobile fractured fragment was fixed with two partially threaded cancellous screws through the lateral approach. At six months follow-up the patient was asymptomatic and fracture had radiologically united.

Jiang *et al* reported a 27-year non-union Hoffa fracture in a 46-year old male^2^. They found intra-operatively a proliferative osteophyte providing sufficient support to the fractured fragment of femoral condyle for the motion of knee during the patient’s routine daily activities. The fracture fragment was fixed with buttress plate along with xenogenous bone graft. At follow-up of the patient at 12 months, he was asymptomatic with knee range of movement 00-1250.

Our case is different from previously reported non-union cases as there was a fibrous non-union at the fracture site, not allowing the fractured fragment to move. Since the fractured fragment was undisplaced and not movable, we fixed the fracture *in situ* without any bone graft. Our patient had full range of knee motion pre-operatively which helped him doing physiotherapy and achieving full movements post-operatively. Our patient became asymptomatic with full range of motion at knee, so there was no reason to get CT scan to see the union status.

In conclusion, we emphasise the possibility of fibrous non-union of Hoffa fracture. This fibrous non-union, if un-displaced, can be treated with *in situ* fixation without bone grafting.
